# A novel approach for correction of crosstalk effects in pathway analysis and its application in osteoporosis research

**DOI:** 10.1038/s41598-018-19196-2

**Published:** 2018-01-12

**Authors:** Yu Zhou, Yunlong Gao, Chao Xu, Hui Shen, Qing Tian, Hong-Wen Deng

**Affiliations:** 1 Center of Genomics and Bioinformatics, Tulane University, New Orleans, LA 70112 USA; 2 Department of Cell and Molecular Biology, Tulane University, New Orleans, LA 70118 USA; 3 Department of Biostatistics and Bioinformatics, Tulane University, New Orleans, LA 70112 USA

## Abstract

Osteoporosis is a prevalent bone metabolic disease and peripheral blood monocytes represent a major systemic cell type for bone metabolism. To identify the key dysfunctional pathways in osteoporosis, we performed pathway analyses on microarray data of monocytes from subjects with extremely high/low hip bone mineral density. We first performed a traditional pathway analysis for which different pathways were treated as independent. However, genes overlap among pathways will lead to “crosstalk” phenomenon, which may lead to false positive/negative results. Therefore, we applied correction techniques including a novel approach that considers the correlation among genes to adjust the crosstalk effects in the analysis. In traditional analysis, 10 pathways were found to be significantly associated with BMD variation. After correction for crosstalk effects, three of them remained significant. Moreover, the MAPK signaling pathway, which has been shown to be important for osteoclastogenesis, became significant only after the correction for crosstalk effects. We also identified a new module mainly consisting of genes present in mitochondria to be significant. In summary, we describe a novel method to correct the crosstalk effect in pathway analysis and found five key independent pathways involved in BMD regulation, which may provide a better understanding of biological functional networks in osteoporosis.

## Introduction

Osteoporosis is the most common metabolic bone disease, mainly manifested as low bone mineral density (BMD). One of the key pathophysiological mechanisms of this disease is excessive bone resorption (by osteoclasts) over bone formation (by osteoblasts).

Peripheral blood monocytes (PBMs) are an appropriate cell model for studying osteoporosis^1^. First, PBMs may act as precursors of osteoclasts^2–5^, the bone resorption cells. Particularly for the adult peripheral skeleton (e.g., one of the most important skeletal site - femur), circulating monocytes provide the sole source of osteoclast precursors^6^. Second, PBMs can secrete a number of potent cytokines important for osteoclast differentiation, activation, and apoptosis^7–10^. Reduced production of PBM cytokines represents a major mechanism for the inhibitory effects of sex hormones on osteoclastogenesis and bone resorption^11–13^. With their abundance and diverse roles in bone metabolism, PBMs may thus represent a highly valuable and unique working cell model for dissecting some of the important pathogenesis mechanisms underlying various skeletal disorders. In fact, abnormalities in PBMs, not only by their percentage in circulation but also by their functional activities, have been linked to a variety of skeletal disorders and traits, such as osteoporosis^14^, rheumatoid arthritis^15^ and alcoholism^16^. Therefore, our study will use PBMs as a cell model to investigate the pathways associated with osteoporosis.

In recent years, taking advantage of high-throughput technologies, pathway analyses have been performed as a crucial step in expression profiling studies for osteoporosis, e.g.^17–19^. The majority of these analyses applied typical approaches to identify the pathways related to BMD variation, such as KEGG and Gene Ontology analysis, which treat the pathways as independent. However, because pathways may have regulatory interactions, or some genes may overlap with each other in different pathways, the derivation of p-values which aim to quantify the significance of the involvement of each pathway in a given phenotype will be affected, which may lead to both false positive and false negative results. The term “crosstalk” was coined by Donato *et al*.^20^ to represent the effect that pathways influence each other via overlapping genes. By simulation test, they found that three major pathway analysis methods (Fisher’s exact test, signaling pathway impact analysis and gene set enrichment analysis) produced a significant number of false positives due to crosstalk effects, and that crosstalk could be explained by the presence of overlapping genes among pathways^20^. Thus, they proposed a method called Maximum Impact Estimation based on maximum likelihood (ML) to correct the crosstalk effects by reassigning each gene to only one of the pathways which it originally belongs to.

Inspired by Donato’s study, the goal of this work was to apply crosstalk correction methods to identify the pathways associated with BMD variations. Toward this goal, we detected the existence of crosstalk effects by classical overrepresentation analysis (ORA) and then applied the ML method to correct these crosstalk effects. However, the ML method did not consider correlation among genes. Based on biological perspectives, expression levels of genes in the same pathway most likely are associated and thus correlated with each other. Here, we further propose a novel improved correction approach based on correlation among genes to improve pathway analyses, then compare the results from all the three methods. ML method corrects the crosstalk effects by reassigning the overlap genes to a unique pathway, but it may generate false positive results because it is mainly based on mathematical not biological prediction. Our approach focuses on the interaction between the genes in the same pathway and could further improve the correction for crosstalk effects. With the application of the methods to osteoporosis research, we identified an independent functional module which may play a different role from the pathways they conventionally and originally belong to.

## Results

### Classical ORA and crosstalk effects

After gene expression data processing, 591 genes were identified as nominal DE genes (p < 0.05) in the “core set genes” (n = 22011). Among the core set genes, 4801 genes were present in at least one KEGG pathway and 103 of them were nominal DE genes. Using the classical ORA methods, ten pathways were significantly associated with BMD variation (p < 0.05) (Table 1). The most significant pathway was oxidative phosphorylation (p-value = 0.0018).

**Table 1:** Top 12 significant pathways by ORA analysis (ranked by raw p-values).

Rank	Pathway	P-value
1	Oxidative phosphorylation	0.0018
2	RIG I like receptor signaling pathway	0.0029
3	Glycosphingolipid biosynthesis lacto and neolacto series	0.0174
4	Cytosolic DNA sensing pathway	0.0202
5	Huntington’s disease	0.0215
6	Parkinson’s disease	0.0225
7	Regulation of autophagy	0.0256
8	Alzheimer’s disease	0.0388
9	Fatty acid metabolism	0.0471
10	Epithelial cell signaling in helicobacter pylori infection	0.0476
11	Adipocytokine signaling pathway	0.0523
12	Antigen processing and presentation	0.0548

**Figure 1:**
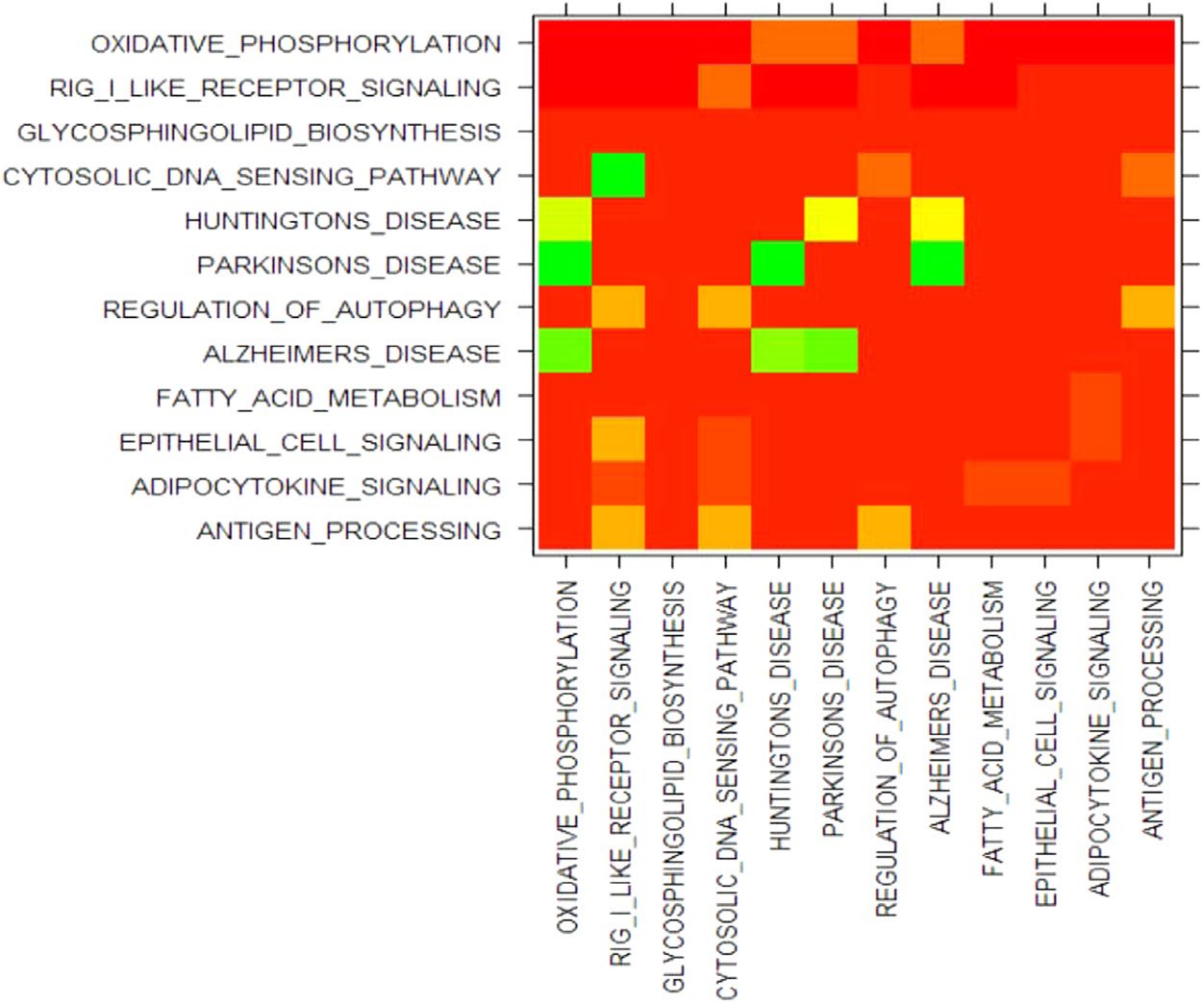
Detail of the crosstalk effect in pathway analysis. The diagonal cells were the original p-values of *P*_*i*_ computed by the classical ORA. The cell [*i, j*] was the p-value of pathway *P*_*i*_ after eliminating the crosstalk effect with *P*_*j*_. The color of each cell represented the p-value: bright red for p-values close to zero, bright green for p-values close to 1.

Figure 1 shows the crosstalk effects in the top 12 pathways (ranked by the raw p-values) for BMD variation. The oxidative phosphorylation pathway became non-significant when the overlapping genes with any degenerative diseases of the central nervous system (Huntington’s, Parkinson’s, or Alzheimer’s disease) were removed (row 1, column 5/6/8). Meanwhile, the significance of these three central nervous system diseases disappeared when their crosstalk effects with oxidative phosphorylation pathway were eliminated (row 5/6/8, column 1). The same phenomenon was found among Huntington’s, Parkinson’s and Alzheimer’s disease pathways. The crosstalk effects also influenced the significance of Rig-I-like receptor signaling, cytosolic DNA sensing and regulation of autophagy pathway.

**Figure 2:**
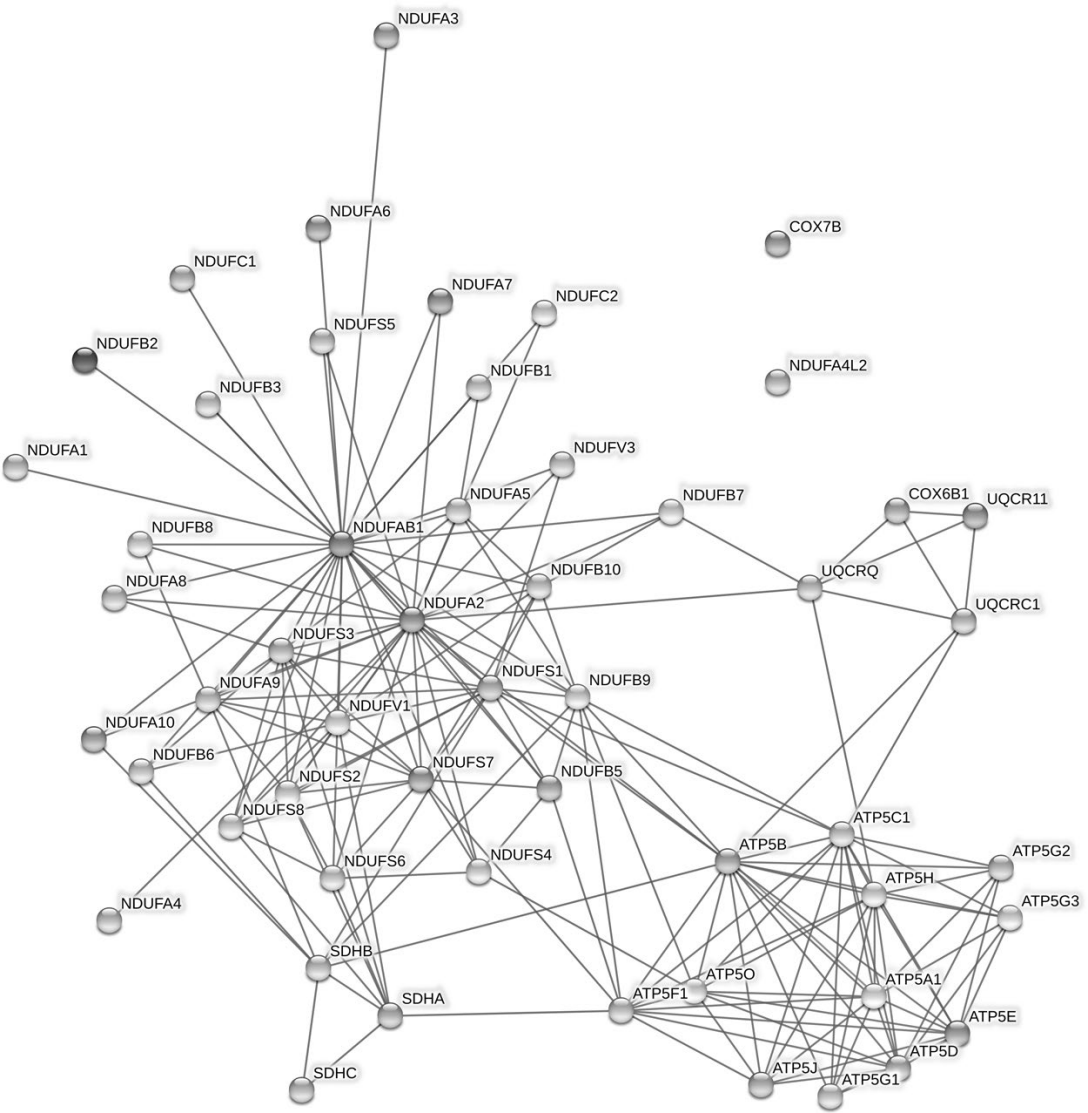
The structure of the Intersection_Pak_Oxi_Hun_Alz module. The edges represented the interaction sourced from experimental evidences.

Via the module detection methods described in Methods section, two new independent modules were generated: 1) Intersection of Alzheimer’s, Parkinson’s, Huntington’s disease and oxidative phosphorylation pathway (Intersection_Pak_Oxi_Hun_Alz); and 2) Intersection of Rig-I-like receptor signaling, cytosolic DNA sensing and regulation of autophagy pathway (Intersection_Rig_Cyt_Auto). A total of 53 genes were included in the module, Intersection_Pak_Oxi_Hun_Alz (Fig. 2), which mainly consists of two gene families: the NADH:ubiquinone oxidoreductase (NDUF) family and the ATP Synthase family. These two gene families are mainly involved in the energy transfer in the mitochondria and thus the new module represents an independent and specific function significance in energy metabolism.

### Crosstalk effects correction by ML method

The ML method was performed to uniquely reassign the genes to pathways and correct the crosstalk effects. The pathways that were found significant after correction are listed in Table 2. Among the 10 significant pathways identified by the classical ORA method, only the pathways for fatty acid metabolism and glycosphingolipid biosynthesis lacto and neolacto series, which were identified significant in Table 1, remain significant after the correction of crosstalk effects. Also, instead of the individual pathways for Alzheimer’s, Parkinson’s, Huntington’s disease and oxidative phosphorylation, the new module, Intersection_Pak_Oxi_Hun_Alz, showed significant association with BMD variation after the correction. Furthermore, seven pathways (Leishmania infection, Adipocytokine signaling pathway, Chemokine signaling pathway, Glycosaminoglycan biosynthesis chondroitin sulfate, JAK-STAT signaling pathway, Natural killer cell mediated cytotoxicity, Protein export) which had not been significant in the classical ORA became significant and were considered to be associated with BMD variation after crosstalk effects correction.

**Table 2:** Top 12 significant pathways by ML method (ranked by raw p-values).

Rank	Pathway	P-value
1	Fatty acid metabolism	0.0005
2	Leishmania infection	0.0005
3	Adipocytokine signaling pathway	0.0007
4	Intersection_pak_oxi_hun_alz	0.0042
5	Glycosphingolipid biosynthesis lacto and neolacto series	0.0124
6	Chemokine signaling pathway	0.0256
7	Glycosaminoglycan biosynthesis chondroitin sulfate	0.0351
8	JAK-STAT signaling pathway	0.0351
9	Natural killer cell mediated cytotoxicity	0.0411
10	Protein export	0.0450
11	GNRH signaling pathway	0.0630
12	Viral myocarditis	0.0831

### Crosstalk effects correction by PCA (principle component analysis) method

After correcting crosstalk effects via the PCA method, the number of significant pathways was much lower than for the ORA results or the ML method results (Table 3). The module Intersection_Pak_Oxi_Hun_Alz exhibited the strongest association with BMD (p = 0.0052). The fatty acid metabolism and glycosphingolipid biosynthesis lacto and neolacto series pathways were confirmed to be significant after the correction by the PCA method. In addition, the MAPK signaling pathway was identified as significant only by the PCA method, but not by the other two methods.

**Table 3:** Top 12 significant pathways by PCA method (ranked by raw p-values).

Rank	Pathway	P-value
1	Intersection_Pak_Oxi_Hun_Alz	0.0052
2	Glycosphingolipid biosynthesis lacto and neolacto series	0.0062
3	Cytosolic DNA sensing pathway	0.0117
4	Fatty acid metabolism	0.0149
5	MAPK signaling pathway	0.0335
6	Glycosaminoglycan biosynthesis chondroitin sulfate	0.0558
7	Protein export	0.0615
8	Leishmania infection	0.0630
9	Peroxisome	0.1042
10	Natural killer cell mediated cytotoxicity	0.1132
11	Intestinal immune network for IGA production	0.1221
12	Thyroid cancer	0.1221

### Simulation

In simulations, ORA was designed to yield 100% power, but the type I error (false positive rate) was 5.0%. For ML method, type I error was 4.1% and type II error was 15.6%. For the PCA method, type I error was 1.9% and type II error was 19.4%.

## Discussion

In this study, we aimed to identify the important pathways involved in osteoporosis mechanisms by analyzing transcriptome-wide gene expression of monocytes in 73 Caucasian females with extremely high or low hip BMD. Unlike traditional pathway analysis studies, we adopted a detection and correction approach for crosstalk effects among pathways during the analysis process. Furthermore, we proposed and employed a novel method (PCA) to correct the crosstalk effects based on the correlation of experimental expression data within pathways or the new modules detected and constructed. Since the PCA considered interaction among genes in the same pathway, it included more information from the experiment, especially the regulatory networks in the pathways, and generated biologically more meaningful results for a better understanding of the pathophysiological mechanisms. Using a module detection algorithm and the correction methods described in Methods section, we found that two pathways were persistently significant in all the results, and importantly, we identified a new independent functional module underlying BMD variation.

The classical ORA and other prevalent pathway analyses treat pathways as independent^20^. However, pathways in the majority of pathway databases may share genes with each other. Genes may participate in different pathways, leading to non-independence among pathways; this is essentially a mathematical/statistical problem that will lead to undesired false positive/negative results. Donato *et al*.^20^ have shown that the traditional pathway analysis approaches produced a significant number of false positives due to crosstalk effects. In our ORA results, RIG I like receptor signaling pathway was identified as the second most significant pathway underlying osteoporosis. However, there is no literature showing the relationship between them. Through the heat map, we observed that four pathways (Alzheimer’s, Parkinson’s, Huntington’s disease and oxidative phosphorylation pathway) lost their significance when the overlapping genes between any two of them were eliminated. This result suggested that the intersection of these pathways determined their significance. Our follow-up analysis further showed that these genes should be considered as an independent functional module.

To correct the crosstalk effects, Donato *et al*.^20^ provided an approach using maximum impact estimation based on maximum likelihood. We also applied this approach to analyze our dataset. However, the results were not as good as its applications in other experiments^20^, yielding results that did not make sense biologically. For example, Leishmania infection describes the pathway underlying a disease spread by the bite of certain types of sandflies. It is unlikely to be related to osteoporosis and no study found any connection between them. However, it became the second most significant pathway after the crosstalk correction using ML.

When a gene is involved in a pathway, its expression levels may affect the expression levels of other genes or be affected by other genes in the same pathway. Based on this biologically realistic consideration, our PCA method reassigned a given gene to the pathway where it has the strongest connection with the rest of the genes. Compared with the ORA or ML results, all the biologically unlikely pathways were excluded from the significant list via PCA correction based on the gene expression correlations, while the mitogen-activated protein kinase (MAPK) signaling pathway was identified as significantly contributing to BMD variation. The MAPK signaling pathway plays important roles in both osteoblast and osteoclast biology. In particular, for osteoclast differentiation from PBM, several studies have confirmed that a p38 MAPK inhibitor, SB203580, could also inhibit RANKL-induced osteoclast differentiation^21,22^. Eugenol, another compound which can mediate attenuation of RANKL-induced NF-κB and MAPK pathways, could synergistically contribute to the inhibition of osteoclast formation^23^. Two pathways, fatty acid metabolism and glycosphingolipid biosynthesis lacto and neolacto series, were still significant. Essential fatty acid (EFA)-deficient animals have been shown to develop severe osteoporosis^24^, while inhibition of glycosphingolipid synthesis has been proven to affect osteoclastogenesis and reduce osteoclast activation^25^.

Although links between osteoporosis and Alzheimer’s^26^, Parkinson’s^27^ and Huntington’s^28^ disease have been reported, the underlying molecular mechanisms are still unclear. Our new module, which mainly consisted of protein families present in mitochondria, was consistently significant for both correction methods. This result suggests that mitochondrial activity may play a key role in the relevance of these diseases to osteoporosis. A recent study indicated that deletion of NDUFS4 may promote osteoclast differentiation and bone resorption via both cell-autonomous and systemic regulation^29^.

We performed the simulation by the process reported in Donato’s paper^20^. Compared with the ORA and ML methods, the PCA method has the smallest type I error (1.9%). It indicates that PCA method could significantly reduce the false positive rate. In simulation or real situation, the number of true non-significant pathways would be much larger than the number of true significant pathways. So the small increase of false positive rate will remarkably amplify the number of falsely detected significant pathways. Although the PCA method had higher type II error than ML method (19.4% vs 15.6%), the commonly used threshold of type II error used in randomized clinical trials was 20%^30–32^, which was higher than the PCA results. Therefore, the type II error rate of the PCA method is still reasonable and acceptable.

There are several limitations of this study. First, PBMs are not equal to osteoclasts. Our results could imply the pathophysiological mechanism of osteoporosis, but the findings need further direct validation. Second, the current knowledge about signaling pathways are still lacking. In our case, only ~4800 genes out of 22,000 genes (microarray data) were able to be annotated to the KEGG pathways. Third, in our method, the construction of new modules was based on the Jaccard Similarities between the new modules. In current study, the Jaccard Similarities were either 0 or greater than 0.8. Therefore, we merged two modules which have a non-zero Jaccard Similarity into a new module. But the Jaccard Similarities could be more various in other studies. Then, the threshold should be set to identify the “similar” new modules. The new modules will be merged in to a new module when their Jaccard Similarity is greater than the threshold, if not, they will be considered as separate ones in the following analysis processes.

In summary, we performed pathway analysis on gene expression data of monocytes for osteoporosis and detected the crosstalk effects among pathways. To correct the crosstalk effects, we applied a novel method based on the correlation of gene expression levels to reduce false positive results and obtained a better understanding of biological networks underlying osteoporosis.

## Materials and Methods

All the methods were conducted in accordance with the rules and guidelines of the Institutional Review Boards of University of Missouri Kansas City and Tulane University. The Institutional Review Boards of University of Missouri Kansas City and Tulane University approved the study. Written informed consent was obtained from all participants before inclusion in the study.

### Subjects and BMD measurements

Subjects for the study came from our microarray-based transcriptome-wide profiling research of PBMs in 73 Caucasian females with extremely high vs. low hip BMD^33^. (High BMD group: Z_BMD_ > +0.84, n = 42 vs Low BMD group: Z_BMD_ < −0.52, n = 31). Strict exclusion criteria were used to exclude individuals with diseases that might affect bone metabolism.

The hip BMD (g/cm^2^) of each subject was measured using a Hologic dual energy x-ray absorptiometer (DXA) scanner, Hologic QDR-4500 (Hologic Corp., Waltham, MA). The machine was calibrated daily. The coefficient of variation of the DXA measurements for BMD was 0.9%. The obtained BMD value was then transformed into a Z score, which is the number of standard deviations a subject’s BMD differs from the mean BMD of a healthy, ethnic-, gender-, and age-matched reference population. The detailed characteristics of subjects are shown in Table 4 and the early study^33^.

**Table 4:** Basic characteristics of subjects for monocyte microarray analyses.

Menopausal status	High BMD			Low BMD		
	N	Age	Hip BMD Z score	N	Age	Hip BMD Z score
Premenopausal	16	51.0 (1.8)	1.54 (0.52)	15	50.0 (2.0)	−0.93 (0.36)
Postmenopausal	26	54.0 (1.8)	1.28 (0.46)	16	52.6 (2.5)	−1.17 (0.60)
Total	42	52.9 (2.3)	1.38 (0.49)	31	51.4 (2.6)	−1.05 (0.51)

Note: Age and hip BMD Z score are shown as mean (standard deviation).

### Experimental procedures

To generate the expression profiles, PBMs were isolated from whole blood using a monocyte-negative isolation kit (Miltenyi Biotec Inc, Auburn, CA) following the manufacturer’s recommendation. Then, total RNA from monocytes was extracted using Qiagen RNeasy Mini kit (Qiagen, Inc., Valencia, CA) and we used Agilent Bioanalyzer (Agilent, Santa Clara, CA) to control the RNA quality before each array experiment, where RNA integrity number (RIN) should be no less than 7.0. Preparation of cDNA, hybridization, and scanning of the mRNA expression levels by the GeneChip Human Exon 1.0 ST Array (Affymetrix, Santa Clara, CA) were performed according to the manufacturer’s protocol. The raw microarray data for this cohort have been submitted to GEO (Gene Expression Omnibus) under the accession number GSE56814.

### Data preparation

For microarray data analysis, all raw CEL files were imported and processed by the Affymatrix analysis tools in oligo package (version 1.14.0) in R (version 2.3.0). The probe IDs were annotated with their corresponding official gene symbols via annotation file (pd.huex.1.0.st.v2). Due to the higher level of evidence supporting the existence of a particular exon, we only analyzed “core” (annotated by RefSeq) probesets and we excluded the probesets without gene symbol annotation. When multiple probe IDs were matched to the same gene symbol, the probe ID with the lowest p-value was selected to represent that gene. Robust multiarray average method^34^ was applied to normalize the array signals^35^ and differential expression analysis was performed using Student’s t-test through the Bioconductor’s (version 2.7) LIMMA (linear models for microarray data) package (version 3.6.9)^36,37^. Because the sample size was limited (although still among the largest of such studies in the field), we used raw p-value < 0.05 as threshold for nominally significant differential expression (supplementary Table 1).

### Membership matrix preparation

In this study, we constructed a dataset which represents the intersection of genes present in at least one KEGG pathway and the genes measured by the GeneChip Human Exon 1.0 ST Array. In total, we obtained 186 pathways and 4801 genes that overlapped between KEGG dataset (c2.cp.kegg.v4.0.symbols.gmt) and our microarray dataset for further pathway analysis. In these genes, 103 were identified as differentially expressed (DE) genes (at the nominal significance level of p < 0.05) by the methods described above.

With the information from the KEGG database, we established a (*m* + *n*)*k membership matrix *X* (Table 5), where columns represent pathways (*k* is the number of pathways, *k* = 186) and rows represent genes (*n* is the number of DE genes, *n* = 103; *m* is the number of non-DE genes, *m* = 4698). In the matrix, genes are ranked in ascending order of p-values from the differential expression analysis. The top *n* (*n* = 103) rows represent DE genes with p-values < 0.05. The *m* rows represent non-DE (NDE) genes. So for each cell *X*_*i,j*_ of matrix *X*, if gene *i* is included in pathway *j*, *X*_*i,j*_ = 1; otherwise *X*_*i,j*_ = 0.

**Table 5:** Example of a (*m* + *n*)*k membership matrix *X*.

	*P*_*1*_	*P*_*2*_	*P*_*3*_	…	*P*_*k*_
*g*_1_	0	0	0	…	1
*g*_2_	1	1	1	…	0
*g*_3_	0	1	0	…	0
⋮	⋮	⋮	⋮	…	⋮
*g*_*n−1*_	1	0	1	…	0
*g*_*n*_	1	1	0	…	1
*g*_*n* + *1*_	0	0	1	…	0
⋮	⋮	⋮	⋮	…	⋮
*g*_*n* + *m*_	1	0	1	…	0

### Pathway analysis by classical overrepresentation approach

We performed classical overrepresentation analysis (ORA) using Fisher’s exact test to assess the significance of each pathway. For example, Table 6 is the contingency table used to compute the significance of pathway *P*_*i*_. *a*_*i*_ represents the number of DE genes present in pathway *P*_*i*_ (count of 1 s in column *i* from row 1 to row 103); *b*_*i*_ represents the number of NDE (non-differentially expressed gene) genes in pathway *P*_*i*_ (count of 1 s in column *i* from row 104 to row 4801). The result generated by ORA indicates the probability of the number of DE genes contained in pathway *Pi* being equal to or higher than expected by chance.

**Table 6:** The contingency table used to compute the significance of pathway *P*_*i*_.

	*P*_*i*_	${P}_{i}^{c}$	Total
*DE*	*a*_*i*_	*n* − *a*_*i*_	*n*
*NDE*	*b*_*i*_	*m* − *b*_*i*_	*m*
*Total*	*a*_*i*_ + *b*_*i*_	*(n* + *m*) − *(a*_*i*_ + *b*_*i*_*)*	*n* + *m*

### Crosstalk effect test

To test the crosstalk effect in pathway analysis for our dataset, if a pathway *P*_*i*_ shares genes with another pathway *P*_*j*_, we removed the intersection part from *P*_*i*_ and recalculated the significance of the remaining element *P*_*i\j*_ in *P*_*i*_ via ORA. We then used the p-value of *P*_*i\j*_ for each pair of pathways [*i, j*] to establish a k*k matrix, where *k* was equal to the number of pathways and the diagonal cells were the original p-values of *P*_*i*_. Both rows and columns were ordered ascendingly by the original p-values of *P*_*i*_. The crosstalk effects were intuitively shown (Fig. 1) by converting this matrix into a heat map of the negative log p-values.

### Novel module detection

In Fig. 1, we could find that some genes consisted of a module shared by several statistical significant pathways. If we removed this module, these pathways became non-significant. So, the result implied that this module played key function in the disease and was more important than the original designated pathways.

To search the key modules among the pathways sharing some common genes, a novel module detection method was applied to pathway pairs. The process is described in detail in ref.^20^. Briefly, for an arbitrary pair of pathways *P*_*i*_ and *P*_*j*_, a module could be created when all three following conditions are satisfied:

1. Both *P*_*i*_ and *P*_*j*_ should have significant results in ORA
2. Neither *P*_*i\j*_ nor *P*_*j\i*_ should be significant
3. The intersection of the two pathways should have a significant ORA result

For any pair of modules *M*_*i*_ and *M*_*j*_ (*i* ≠ *j*), which have large Jaccard Similarity, we merged the two modules into a new module. Jaccard Similarity is defined as equation (1).

1 $$mJS=\frac{|{M}_{i}{\cap }^{}{M}_{j}|}{{\rm{\min }}(|{M}_{i}|,|{M}_{j}|)}$$

All the Jaccard Similarities we calculated are either 0 or a value greater than 0.8; therefore, when any two modules have a non-zero Jaccard Similarity, they were merged into a new module.

The new modules were then removed from the pathways with which they overlap. The newly created modules as such and 186 KEGG pathways with new modules excluded were analyzed by crosstalk correction methods in the following. After module detection, membership matrix was expanded to 188 columns, with Column 187 and Column 188 representing new modules.

### Crosstalk correction methods: maximum impact estimation based on maximum likelihood and principle component analysis

#### Maximum Likelihood (ML) method

Donato *et al*.^20^ developed this algorithm, aimed at establishing an underlying pathway impact matrix where each gene contributes to one and only one pathway to correct the crosstalk effects. They named this matrix *Z* as maximum impact matrix and matrix *Z* had the same structure with the membership matrix *X*. But in matrix *Z*, for each gene *i*, one row *Zi* had only a one in column *j (Z*_*i,j*_ =1*)* and zeros elsewhere. It represented that gene *i* had the strongest influence on pathway *j* than on other pathways which also included gene *i*. If there were no crosstalk effect, the matrix *X* and the matrix *Z* would be equal.

The authors used a likelihood-based estimation to calculate the similarity between the matrix *Z* and observing membership matrix *X*. They provided an expectation maximization approach to maximize the similarity by an iterative algorithm. The details of this method were shown in the ref.^20^. Finally, ORA was conducted on maximum impact matrix *Z* instead of membership *X* after matrix *Z* was established.

#### Principle Component Analysis (PCA) method

Instead of calculating the maximum likelihood of the observed membership matrix, our PCA method takes into account the association among mRNA expression levels based on real experimental data, which are more likely to be biologically meaningful and realistic.

We established a matrix of mRNA expression data, where columns represent subjects and rows represent genes. For each pathway *j* (1 <  = *j* <  = *k*, after module selection, *k* = 188 in our study), the following process was conducted:

1. Select all the rows of the genes in pathway *j* and construct a new matrix *E*_*j*_.
2. Create a *(m* + n)*k matrix *C* (with the same size as the membership matrix after module selection) to record the correlation coefficients. Each cell of matrix *C*, *C*_*i,j*_ is computed as follows:
  - If gene *i* is not included in pathway *j*, *C*_*i,j*_ = 0; otherwise:
  - If gene *i* is included in pathway j, remove gene *i* from matrix *E*_*j*_. Use *E*_*j/i*_ to denote the rest of the matrix.
  - Conduct PCA on matrix *E*_*j/i*_ to compute first principle component (PC1) of *E*_*j/i*_, whose length should be equal to the column number of *E*_*j/i*_.
  - Compute the Pearson correlation coefficient between PC1 of *E*_*j/i*_ and each row of gene *i*, which was removed from *E*_*j*_ previously. Assign the greatest correlation coefficient to *C*_*i,j*_.
3. After obtaining matrix *C*, we create the maximum impact matrix *Z* by PCA method. We assume the correlation coefficients between gene *i* and matrix *E*_*j/i*_ should reflect the contribution of gene *i* to pathway *p*. Therefore, *Z*_*i,p*_ = 1 if *C*_*i,p*_ = max{*C*_*i,j│*_1 ≤ *j* ≤ *k*}, otherwise *Z*_*i,p*_ = 0.
4. Conduct ORA on matrix *Z*.

In this method, via PCA on expression data of the rest genes in pathway *j*, we used the first principal component *E*_*j\i*_ to represent pathway *j* without gene *i*. When the P value for correlation between *E*_*j\i*_ and expression level of gene *i* is the lowest, we assumed that it means gene *i* has the highest association with pathway *j* and assigned gene *i* to pathway *j*.

### New module structure

To explore and visualize the biological relationships among genes in the new module detected, STRING v10.0 software was used to build the topological structure of the new module^38^. All the parameters were set to the default values and the interaction between genes were only sourced from experiments.

### Simulation process

Because PCA considered the correlation between genes, we used real data described above in membership matrix preparation as the reference set (including 4801 genes) for simulation analysis. We conducted the simulation according to that described in Donato’s paper^20^. Briefly, for each pathway *P*_*i*_, we calculated the number *n*_*i*_ of DE genes that would make *P*_*i*_ significant by Fisher Exact Test. We simulated a situation as following. There are 100 DE genes in the reference set and *n*_*i*_ of them belong to *P*_*i*_. So pathway *i* should be significant in Fisher Exact Test. In this case, we used the reference set to randomly pick *n*_*i*_ genes from *P*_*i*_ and 100- *n*_*i*_ genes that are not in *P*_*i*_, and calculated the Fisher Exact Test significance of all other pathways. Since the 100- *n*_*i*_ genes that are not in *P*_*i*_ are randomly chosen from the reference set, no other pathway should be significant. In this simulation, the hypothesis is true for the *P*_*i*_, while the null hypothesis is true for all other pathways. We repeated this simulation 1,000 times for each pathway *P*_*i*_, and each time we applied the ORA, ML and PCA methods to calculate the significance of all the pathways.

## Electronic supplementary material

supplementary table 1
